# Transient increase in mitochondrial respiration in blood cells from breast cancer patients following chemo- and radiotherapy

**DOI:** 10.1007/s10238-025-01665-4

**Published:** 2025-05-08

**Authors:** Marie-Louise Abrahamsen, Ida Bager Christensen, Linda Laizāne, Haboon Ismail Ahmed, Kristian Buch-Larsen, Djordje Marina, Michael Andersson, Peter Schwarz, Flemming Dela, Linn Gillberg

**Affiliations:** 1 Xlab, Department of Biomedical Sciences, University of Copenhagen, Blegdamsvej 3B, 2200 Copenhagen, Denmark; 2 Laboratory of Sports and Nutrition Research, Riga Stradiņš University, Riga, Latvia; 3 Department of Nephrology and Endocrinology, Rigshospitalet, Copenhagen, Denmark; 4 Department of Oncology, Rigshospitalet, Copenhagen, Denmark; 5 Faculty of Health and Medical Sciences, University of Copenhagen, Copenhagen, Denmark

**Keywords:** Breast cancer, Adjuvant therapy, High-resolution respirometry, Mitochondria, Peripheral blood mononuclear cells, Inflammation

## Abstract

**Abstract:**

Mitochondrial respiration in peripheral blood mononuclear cells (PBMCs) has previously been shown to increase after chemo- and radiotherapy in early-stage breast cancer (BC) patients, but the persistence of the increase remains unknown. This study assessed whether changes in mitochondrial respiration and content in PBMCs from postmenopausal BC patients persist up to 1 year after treatment. Thirty-four early-stage BC patients were studied before, shortly after, and six- and twelve-months post-treatment along with 20 healthy controls. Mitochondrial respiration was measured using high-resolution respirometry of intact and permeabilized PBMCs. Mitochondrial content was estimated by quantifying mitochondrial DNA relative to nuclear DNA via qPCR. The mitochondrial respiratory capacity of intact and permeabilized PBMCs from BC patients significantly increased after adjuvant chemo- and radiotherapy (+ 33% and + 30% for the maximal capacity of the electron transport system, ETS), consistent with previous findings. Importantly, the respiratory capacity returned to pre-treatment levels six months after treatment completion in both intact and permeabilized cells (− 23% and − 26% for the ETS). Healthy controls exhibited similar mitochondrial respiration but had increased mitochondrial content (+ 20%) compared to BC patients before treatment. In summary, chemo- and radiotherapy transiently increased mitochondrial respiration in PBMCs, returning to baseline within six months after treatment completion. This temporary rise in oxygen demand may reflect immune system activation.

**Graphical Abstract:**

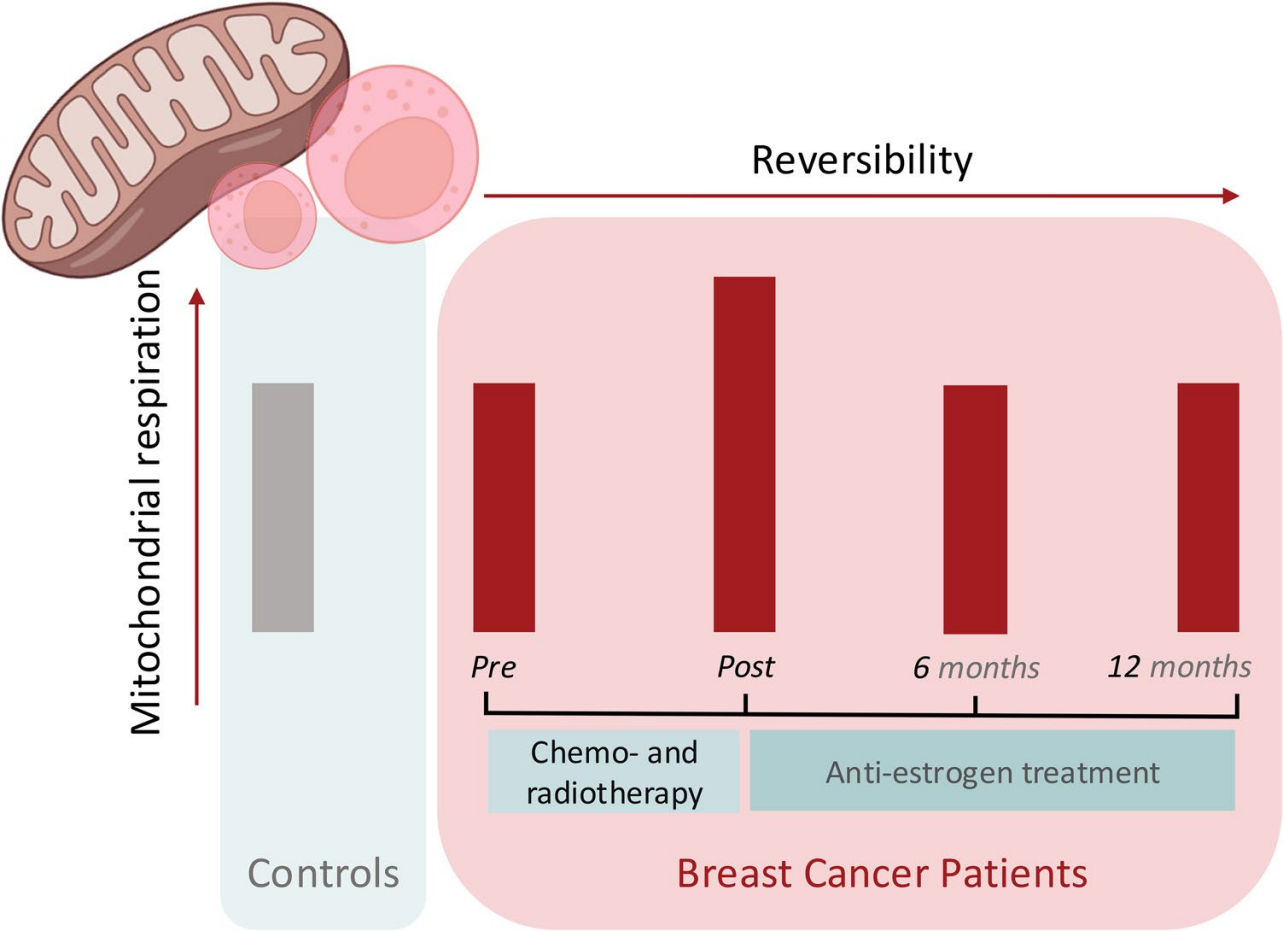

**Supplementary Information:**

The online version contains supplementary material available at 10.1007/s10238-025-01665-4.

## Introduction

Breast cancer (BC) is the most common cancer worldwide with over two million new incidents each year [1]. Multimodal adjuvant treatments, including chemotherapy, radiotherapy, and anti-estrogen treatment, have contributed to increased survival rates. However, the growing group of cancer survivors often experience treatment-related side effects including neuropathy [2], dyslipidemia [3, 4], and systemic inflammation [5] which increases their risk of type 2 diabetes and cardiovascular disease [6, 7]. Although it is well documented that BC patients experience metabolic and inflammatory side effects after adjuvant therapy, what happens on the molecular level remains largely unknown.

Mitochondrial function plays a crucial role in cellular metabolism, and previous studies have demonstrated that chemotherapy can lead to a significant reduction in mitochondrial respiration in skeletal muscle from both rodents [8, 9] and BC patients [10]. Altered mitochondrial respiration might be caused by changes in the mitochondrial content, and previous studies have shown that the mitochondrial content is reduced in skeletal muscle from BC patients after chemotherapy compared to before [10–12] and was lower after chemotherapy compared to healthy controls [13]. In contrast to the findings in skeletal muscle [10], our group previously demonstrated that mitochondrial respiration and content in peripheral blood mononuclear cells (PBMCs) from BC patients significantly increase with adjuvant chemo‐ and radiotherapy [14]. However, none of the previous studies investigated whether therapy-induced changes persist or reverse within the first months or years post-treatment.

In this study, we investigated mitochondrial respiratory capacity and content in PBMCs from BC patients, tracked from before chemo‐ and radiotherapy to 1 year post-treatment, and compared the data with that of age- and BMI-matched healthy controls.

## Materials and methods

### Clinical study

The clinical study has previously been described [3]. Postmenopausal women with early-stage (non-metastatic) BC were recruited to the clinical study *Healthy Living after Breast Cancer* (NCT03784651) at the Department of Oncology, Rigshospitalet, Denmark [3]. We evaluated 34 BC patients before (pre), shortly after (post), and six and twelve months after chemo- and radiotherapy. Patients with previous malignancies or preexisting endocrine or metabolic diseases were excluded. Anthropometric measurements and blood sampling were performed at the research unit of the Department of Nephrology and Endocrinology, Rigshospitalet, Denmark. Twenty-four BC patients received chemotherapy after tumor resection (adjuvant), whereas ten patients received chemotherapy before surgery (neoadjuvant) (Supplementary Table 1). The post-treatment blood sampling was taken on average 77 days after completion of chemotherapy. Most patients (74%) received a combination of paclitaxel, cyclophosphamide, and epirubicin as (neo)adjuvant chemotherapy. In addition, 85% of the BC patients received radiotherapy sequentially after chemotherapy, and 85% had ER + disease and were treated with anti-estrogen therapy, which was initiated before the post-treatment blood sampling. None of the BC patients had triple-negative disease. A control group of 20 BMI- and age-matched healthy controls were also included in the study and had anthropometric measurements and blood samples taken at the research unit of the Department of Nephrology and Endocrinology, Rigshospitalet, Denmark.

### High-resolution respirometry analyses of PBMCs

The procedure for PBMC isolation has previously been described [14]. In brief, isolated PBMCs were re-suspended in Mitochondrial Respiration media 05 (MiR05). The concentration and viability of the isolated PBMCs were quantified using the NucleoCounter NC-3000, the staining reagent Solution 13 (ChemoMetec), and the Cell count and viability assay (NucleoView NC-3000 software, ChemoMetec). The oxygen consumption of isolated PBMCs was measured by high-resolution respirometry (HRR) using Oxygraph-O2k instruments (Oroboros Instruments) and the DatLab 6.1.0.7 software (Oroboros Instruments) which has previously been described in detail [14]. For each participant sample, 1.5 million PBMCs were added to each Oxygraph-O2k chamber and the oxygen consumption rate per million PBMCs was determined in duplicate for both intact and permeabilized PBMCs using the following HRR protocols:

*Intact cells*: (1) Endogenous routine respiration (before the addition of any substrate to the chamber); (2) Oligomycin (4 µg/mL) addition to inhibit the phosphorylation pathway (ATP synthase) and enable the measurement of LEAK state respiration; (3) Titration of FCCP (0.25 µM, until decline of O_2_ flux) to collapse the proton gradient and measure the maximal capacity of the ETS during uncoupled respiration; (4) Rotenone (0.5 µM) addition to inhibit complex I; and (5) Antimycin A (2.5 µM) addition to inhibit complex III and enable measurement of non-mitochondrial respiration.

*Permeabilized cells*: (1) Endogenous routine respiration; (2) Digitonin (5 µg/mL DMSO) addition to permeabilize the plasma membrane of the cells; (3) Malate (2 mM), Glutamate (10 mM), and Pyruvate (5 mM) addition to determine complex I-simulated LEAK respiration; (4) ADP (5 mM) and MgCl_2_ (3 mM) addition to stimulate the ATP synthase and estimate state 3 respiration with complex I-linked substrates (CI_*P*_); (5) Cytochrome c (0.01 mM) addition to evaluate the intactness of the outer mitochondrial membrane; (6) Succinate (10 mM) addition to measure state 3 respiration with complex I and II-linked substrates (CI + CII_*P*_); (7) Titration of FCCP (0.25 µM, until the decline of O_2_ flux) to collapse the proton gradient and measure the maximal capacity of the ETS during uncoupled respiration; (8) Rotenone (0.5 µM) addition to inhibit complex I; and (9) Antimycin A (2.5 µM) addition to inhibit complex III and enable measurement of non-mitochondrial respiration.

Based on the HRR results, the respiratory control ratio (RCR) was assessed to evaluate mitochondrial efficiency (i.e., capacity for substrate oxidation and ATP turnover) [15]. For the intact cells, the RCR was calculated as the ratio between uncoupled respiration (ETS, state 3u) and Oligomycin inhibited respiration (proton leak, state 4o) [15]. For the permeabilized cells, RCR was calculated as the ratio between maximal ADP-supported respiration (CI + CII_*P*_, state 3_ADP_) and respiration in the absence of ADP (LEAK_CI_, state 4) [15]. In addition, the reserve capacity was calculated to evaluate the mitochondria's ability to adapt to cellular stress and help prevent ATP shortage during periods of acute stress or increased workload [16]. The reserve capacity was calculated by subtracting the endogenous respiration from the maximal oxygen consumption of the ETS during uncoupled respiration (ETS in the figures).

An overview of all HRR data available from BC and control participants is illustrated in Supplementary Fig. 1.

### Relative mitochondrial content (mtDNA/ncDNA)

Mitochondrial content in PBMCs was estimated by measurements of mitochondrial DNA (mtDNA) relative to nuclear DNA (ncDNA), as previously described [14]. Briefly, DNA was extracted using the Mag-Bind Blood and Tissue DNA HDQ 96 Kit (Omega Bio-Tek) and the KingFisher Duo Prime purification system (Thermo Scientific). DNA concentration was determined with Qubit Fluorometric Quantification and dsDNA Broad Range Assay Kit (Thermo Scientific). qPCR was conducted with the QuantiNova SYBR Green PCR kit (Qiagen) on the QuantStudio Real-Time PCT System (Thermo Scientific). qPCR was performed in triplicates for the mitochondrial tRNA^LEU^ gene (Forward primer: 5′-CACCCAAGAACAGGGTTTGT-3′, Reverse primer: 5′-TGGCCATGGGTATGTTG TTAA-3′) and the nuclear beta-2-microglobulin gene (Forward primer: 5′-TGCTGTCTCCATG-TTTGATGTATCT-3′, Reverse primer: 5′-TCTCTGCT CCC CAC CTC TAAGT-3′) [17]. The mtDNA/ncDNA ratio was determined using the ΔΔCt method [18].

### Statistical analysis

Patient data collected before treatment were contrasted to control data using an unpaired t test for parametric data and Mann–Whitney U test for nonparametric data. Data from the four different patient visits were analyzed using mixed effects analysis with the Geisser-Greenhouse correction for variance in combination with Tukey’s test correcting for multiple comparisons. Nonparametric data were ln-transformed before the mixed effects analysis. Relative mitochondrial content data were analyzed with Wilcoxon tests. Differences between groups were significant if the p-value was < 0.05, while borderline significant differences had p-values between 0.05 and 0.10.

## Results

### Clinical data

Baseline characteristics were assessed to ensure comparability between groups. No significant differences were observed in age, weight, BMI, leukocyte count, or hemoglobin concentration between the 34 BC patients before initiating adjuvant therapies and the 20 controls (Table 1). In BC patients, the leukocyte count and hemoglobin concentration decreased after chemo- and radiotherapy (− 19% and − 6%, respectively) but returned to pre-treatment levels at the twelve-month visit (both + 4%; Table 1). Plasma levels of high-sensitivity C-reactive protein (CRP) were available for a subgroup of the participants (controls: n = 7; patients: pre: n = 24, post: n = 23, 6 months: n = 19, 12 months: n = 13) [5] (Table 1). The CRP level was not significantly different between patients before treatment and controls, and the level did not change significantly over time in the BC patients (Table 1).

**Table 1:** Clinical data of BC patients and healthy controls

	Controls n = 20	Pre-treatment n = 34	Post-treatment n = 34	6 months n = 33	12 months n = 34
Age (years)	60 ± 4	59 ± 4	N/A	N/A	N/A
Weight (kg)	78 ± 19	73 ± 15	72 ± 14	N/A	72 ± 15
BMI (kg/m^2^)	27 ± 7	26 ± 5	26 ± 5	N/A	26 ± 6
Leucocytes (10^9^/L)	5.8 ± 1.8	5.9 ± 2.2	4.8 ± 1.6	4.7 ± 1.2*	5.0 ± 1.7
Hemoglobin (mmol/L)	8.3 ± 0.5	8.4 ± 0.8	7.9 ± 1.6*	8.1 ± 0.6	8.2 ± 0.5¤
CRP (mg/L)	1.2 (0.7, 5.2)	1.2 (0.3, 3.5)	1.6 (0.6, 3.2)	1.0 (0.5, 2.6)	0.7 (0.4, 2.2)

Data are presented as mean ± SD for parametric variables and median (25%, 75% interquartile range) for nonparametric variables. One patient missed the six-month visit (n = 33). The CRP measurements [5] were available for a subgroup of the participants (controls: n = 7, pre: n = 24, post: n = 23, 6 months: n = 19, 12 months: n = 13). *6 months* six months after completing chemo- and radiotherapy, *12 months* 12 months after completing chemo- and radiotherapy, *BMI* body mass index, *CRP* C-reactive protein, *N/A* not applicable. *Significantly different from pre-treatment (p < 0.05). ¤Significantly different from post-treatment (p < 0.05)

### Transient increase in mitochondrial respiration of PBMCs after chemo- and radiotherapy in BC patients

**Fig. 1:**
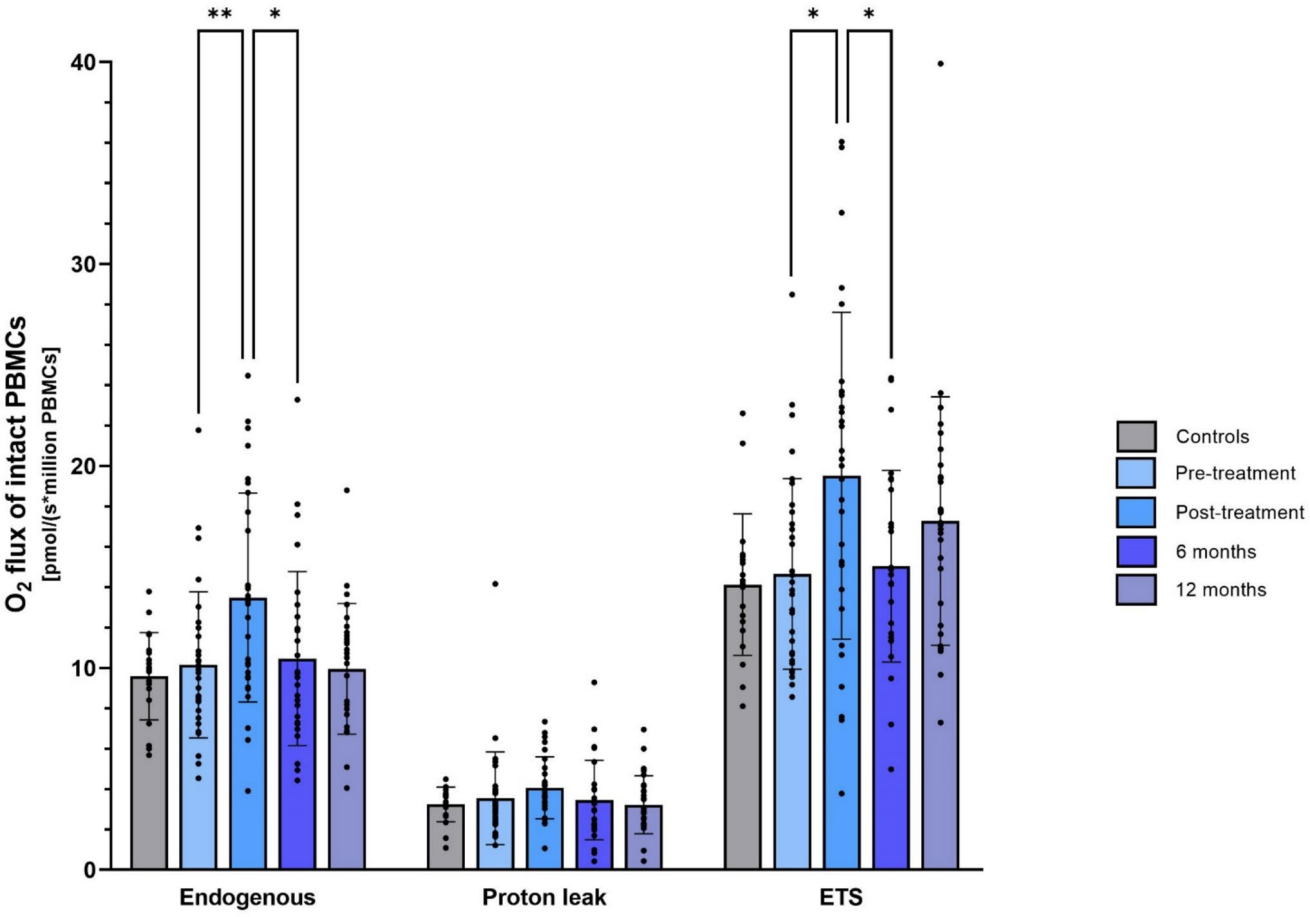
Mitochondrial respiration in intact PBMCs from BC patients and healthy controls. Mitochondrial O_2_ flux in PBMCs from healthy controls (gray bars, n = 20) and BC patients (blue bars) at pre- (n = 32), post- (n = 30), 6-month (n = 28), and 12-month (n = 28) visits after chemo- and radiotherapy. The top of the bar represents the mean, and the error bar shows the standard deviation. Endogenous: Endogenous routine respiration (no substrates or inhibitors added). Proton leak: Oxygen is consumed due to a proton leak over the inner mitochondrial membrane. ETS: Maximal capacity of the electron transfer system. **: p-value < 0.005, *: p-value < 0.05. *BC* breast cancer, *ETS* electron transfer system, *PBMCs* peripheral blood mononuclear cells

**Fig. 2:**
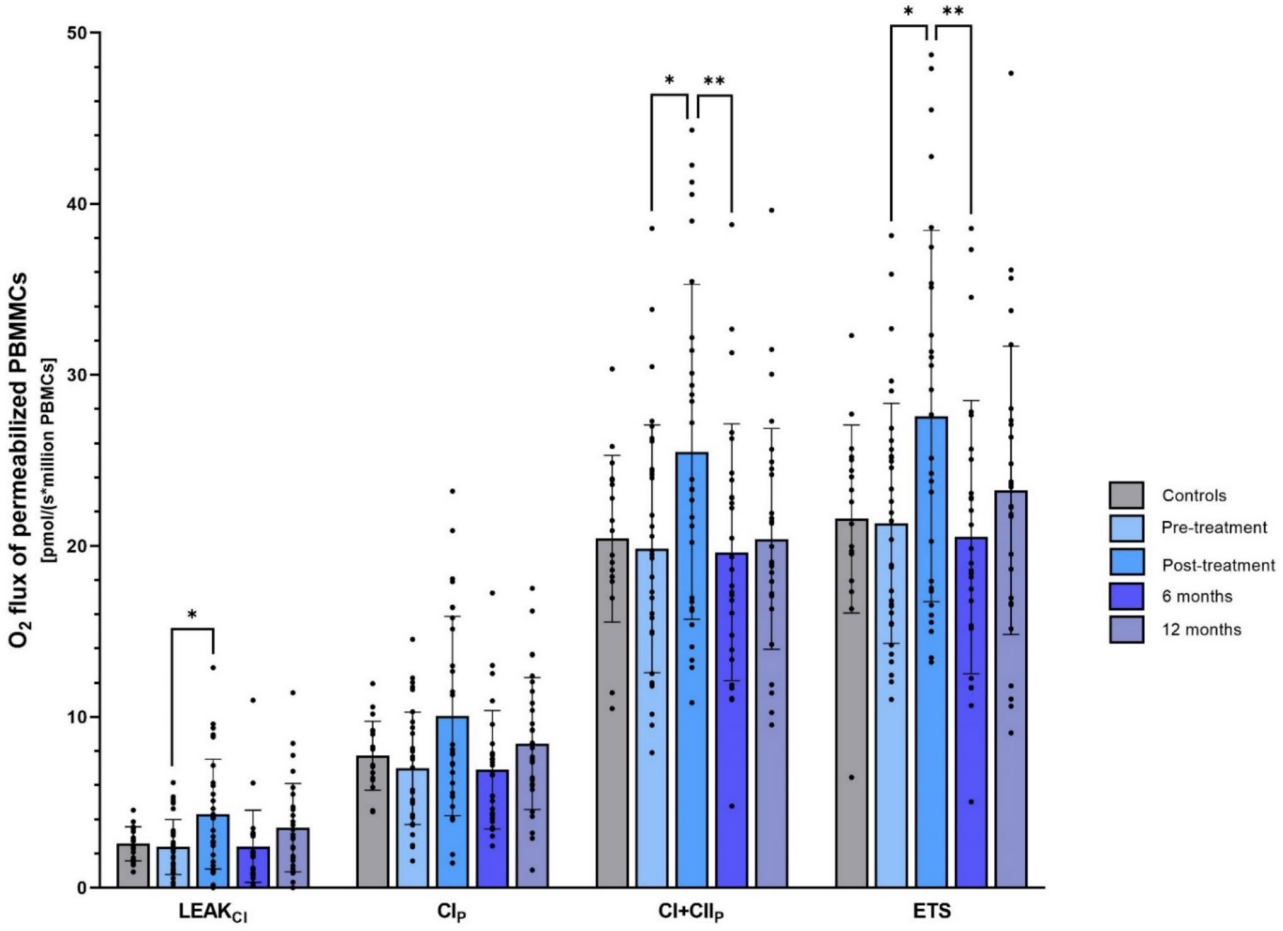
Mitochondrial respiration in permeabilized PBMCs from BC patients and healthy controls. Mitochondrial O_2_ flux in PBMCs from healthy controls (gray bars, n = 19) and BC patients (blue bars) at pre- (n = 32), post- (n = 30), 6-month (n = 28), and 12-month (n = 30) visits after chemo- and radiotherapy. The top of the bar represents the mean, and the error bar shows the standard deviation. LEAK_CI_: Leak respiration in the presence of complex I-linked substrates. CI_P_: Complex I-linked respiration. CI + CII_P_: Complex I + II-linked respiration. ETS: Maximal capacity of the electron transfer system. **: p-value < 0.005, *: p-value < 0.05. *BC* breast cancer, *ETS* electron transfer system, *PBMCs* peripheral blood mononuclear cells

In line with our previous study [14], which included 23 of the 34 BC patients in the current cohort, mitochondrial respiration in PBMCs increased significantly after chemo- and radiotherapy based on HRR analyses of both intact and permeabilized cells (Figs. 1, 2). Specifically, the endogenous routine respiration of intact PBMCs (before the addition of substrates) increased by 32% (p = 0.003, Fig. 1). The maximal capacity of the ETS increased by 33% (p = 0.03), whereas there was no significant change in the oxygen consumed due to proton leak over the inner mitochondrial membrane in intact PBMCs after compared to before chemo- and radiotherapy (p = 0.67, Fig. 1). In permeabilized PBMCs, however, the LEAK respiration in the presence of complex I-linked substrates (LEAK_CI_) increased by 79% after versus before chemo- and radiotherapy (p = 0.01, Fig. 2). The complex I-linked respiration (CI_P_) was borderline significantly increased (43%, p = 0.050), whereas complex I + II-linked respiration (CI + CII_P_) and the maximal capacity of the ETS increased by, respectively, 29% and 30% after chemo- and radiotherapy (p = 0.02 and p = 0.01, Fig. 2).

The unique follow-up samples from the current cohort allowed us to determine the changes in mitochondrial respiration in PBMCs from the BC patients in the time after treatment completion. Interestingly, the endogenous routine respiration in intact PBMCs decreased by 22% six months after chemo- and radiotherapy (p = 0.02, Fig. 1), reaching a level similar to pre-chemotherapy, which remained at a similar level at the twelve-month visit. A similar pattern was seen for the maximal capacity of the ETS in intact cells which decreased by 23% during the six months after therapy completion (p = 0.03, Fig. 1). In permeabilized PBMCs, complex I + II-linked respiration (CI + CII_P_) and maximal capacity of the ETS decreased by, respectively, 23% and 26% during the six months after therapy completion (p = 0.009 and p = 0.008, Fig. 2). Moreover, we found tendencies for decreased LEAK (LEAK_CI_, 44%, p = 0.08) and complex I-linked (CI_P_, 31%, p = 0.054) respiration in permeabilized PBMCs six months after completed chemo- and radiotherapy with no significant changes between the six- and twelve-month visit (Fig. 2). An overview of the mitochondrial changes after chemo- and radiotherapy and subsequent follow-up visits is presented in Supplementary Table 2.

We found no significant changes in mitochondrial efficiency (RCR), in either intact or permeabilized PBMCs for the patients over time (Table 2). The reserve capacity (mitochondria’s ability to meet extra energy requirements) was not significantly different in patients pre- vs post-treatment (Table 2) but was significantly increased at the twelve-month visit compared to the six-month visit in both intact (p = 0.0003) and permeabilized PBMCs (p = 0.046, Table 2). In intact PBMCs, the reserve capacity at the twelve-month visit was also significantly higher than the pre-treatment level (p = 0.01).

**Table 2:** Reserve capacity and respiratory control ratio of intact and permeabilized PBMCs

	Controls	Pre-treatment	Post-treatment	6 months	12 months
**Intact cells**					
RCR	4.0 (3.6, 5.8)	4.7 (3.6, 5.5)	5.0 (3.7, 6.0)	4.5 (3.6, 6.5)	4.0 (4.3, 6.5)
Reserve capacity	4.5 ± 2.6	4.3 ± 2.8	6.0 ± 4.1	4.6 ± 2.9	7.5 ± 3.8*#
**Permeabilized cells**					
RCR	7.5 (6.9, 9.7)	9.0 (6.5, 16.8)	7.2 (5.1, 11.1)	8.0 (6.6, 12.7)	7.1 (4.8, 10.5)
Reserve capacity	11.9 ± 4.1	11.0 ± 4.1	13.5 ± 7.0	10.1 ± 5.6	13.6 ± 5.7#

Respiratory control ratio (RCR) [15] represents the mitochondrial efficiency, whereas the reserve capacity estimates mitochondria’s ability to meet extra energy requirements [16]. Data are presented as mean ± SD for parametric variables and median (25%, 75% interquartile range) for nonparametric variables. Respiratory ratios were calculated for intact and permeabilized PBMCs from healthy controls (n = 20) and BC patients pre- (n = 32), post- (n = 30), 6 months (n = 28), and 12 months (n = 30) after chemo- and radiotherapy. *: Significantly different from pre-treatment visit (p-value < 0.05). #: Significantly different from 6-month visit (p-value < 0.05). *BC* breast cancer, *ETS* electron transfer system, *OXPHOS* oxidative phosphorylation, *PBMCs* peripheral blood mononuclear cells, *RCR* respiratory control ratio

### Mitochondrial respiration in PBMCs from BC patients vs controls and across treatment groups in BC patients

Uniquely for this study, we included a group of 20 healthy age- and BMI-matched controls to investigate whether the BC diagnosis itself might be associated with altered PBMC respirometry. We found no significant differences in any of the mitochondrial respiratory states between the 34 early BC patients and the 20 healthy controls, indicating that BC diagnosis did not influence PBMC respiration in postmenopausal women (Figs. 1 and 2, Table 2, and Supplementary Table 2).

In line with the above results and similar to our previous publication [14], we found that PBMC mitochondrial respiration in BC patients with a breast tumor present at the time of blood sampling (neoadjuvant group, n = 10) was not different from that of patients who had their tumor removed before blood sampling (adjuvant group, n = 24, Supplementary Fig. 2A). However, we observed a 26% higher endogenous routine respiration in intact PBMCs in the neoadjuvant group compared to the adjuvant group *post-*treatment (p = 0.048, Supplementary Fig. 2A).

The endogenous mitochondrial respiration increased post-chemo- and radiotherapy independently of the type and combinations of chemotherapy drugs (Supplementary Fig. 2B). Unfortunately, we lack statistical power to investigate the influence of radiotherapy and anti-estrogen therapy on PBMC mitochondrial respiration in BC patients.

Finally, various mitochondrial respiration states of intact and permeabilized cells were correlated with plasma levels of CRP to investigate whether PBMC respiration was associated with this inflammation marker. We found no correlation between the two measurements (Supplementary Table 3).

### No changes in relative mitochondrial content after vs before adjuvant therapy

**Fig. 3:**
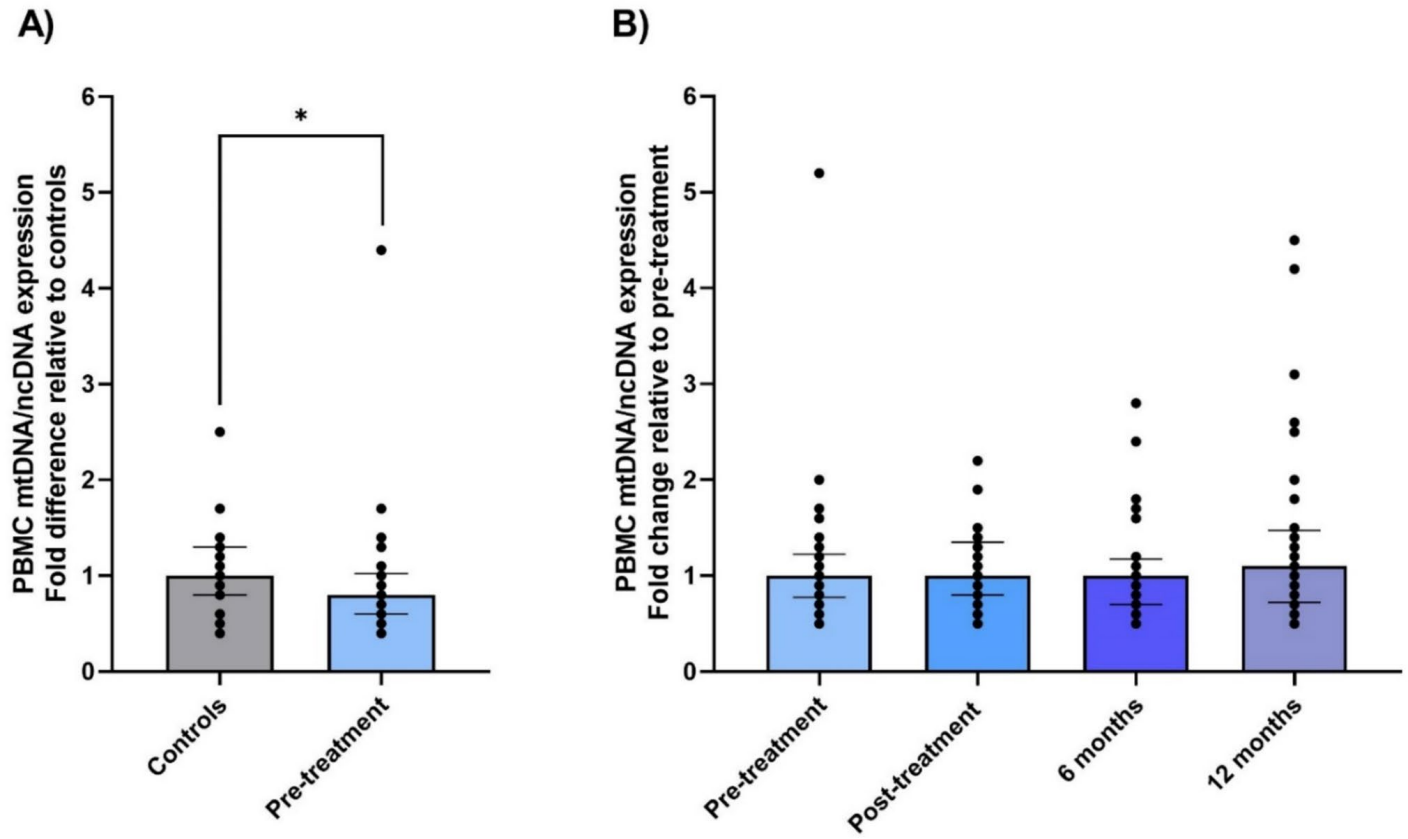
Mitochondrial DNA (mtDNA) relative to nuclear DNA (ncDNA) in PBMCs from BC patients and healthy controls. Relative mitochondrial content estimated with qPCR amplification of the mitochondrial tRNA^LEU^ gene (mtDNA) normalized to the nuclear beta-2-microglobulin gene (ncDNA) for **A** BC patients pre-treatment (n = 34) relative to healthy controls (n = 20), and for **B** BC patients shortly after (post, n = 33) and six (n = 28) and twelve (n = 32) months after chemo- and radiotherapy relative to pre-treatment. The top of the bar represents the median, whereas the error bar shows the interquartile range. *: p-value < 0.05. *BC* breast cancer, *PBMCs* peripheral blood mononuclear cells

The relative mitochondrial content (mtDNA/ncDNA) in isolated PBMCs from patients and controls was significantly lower in BC patients pre-treatment relative to healthy controls (− 20%, p = 0.02, Fig. 3A). We found no significant changes in the relative mitochondrial content of PBMCs in BC patients pre-treatment compared to the later visits (post: p = 0.52, 6 months: p = 0.85, 12 months: p = 0.15, Fig. 3B). The results contrast with our previous study where mtDNA/ncDNA increased from pre- to post-therapy [14].

The relative mitochondrial content correlated significantly with the proton leak (r = 0.29, p = 0.04) and non-mitochondrial respiration (r = 0.37, p = 0.03) in intact cells and with CI_P_ (r = 0.29, p = 0.04) and non-mitochondrial respiration (r = 0.35, p = 0.045) in permeabilized PBMCs (Supplementary Table 4). When correcting the mitochondrial O_2_ flux for mitochondrial content (intrinsic respiration), there were no longer significant differences in PBMC mitochondrial respirometry before compared to after chemo- and radiotherapy (data not shown). Furthermore, the mitochondrial content was borderline significantly higher in the neoadjuvant group compared to the adjuvant group at the post-treatment visit (p = 0.059, data not shown), and the difference in mitochondrial respiration between the adjuvant and neoadjuvant group after chemo- and radiotherapy (Supplementary Fig. 2A) was no longer statistically significant after correction for mitochondrial content.

## Discussion

This study demonstrates for the first time that chemo- and radiotherapy for postmenopausal BC causes a temporary increase in mitochondrial respiration in PBMCs, which returns to pre-treatment levels six months after therapy completion. This indicates that PBMCs from BC patients have an elevated oxygen demand to support cellular function during the initial months following chemo- and radiotherapy treatment. Furthermore, this study shows that mitochondrial respiration is comparable between patients before therapy and postmenopausal women without BC. This suggests that the observed changes in mitochondrial respiration are primarily driven by chemo- and radiotherapy, as evidenced by their reversibility within the first year after treatment completion [14].

We observed no significant changes in relative mitochondrial content (mtDNA/ncDNA) in PBMCs from BC patients after compared to before adjuvant therapy, in contrast to our previous study [14]. Based on the correlations between the relative mitochondrial content and several of the respiratory states, along with the absence of significant therapy-induced changes in intrinsic respiration (mitochondrial respiration adjusted for mitochondrial content), alterations in mitochondrial content may account for some of the observed changes in mitochondrial respiration.

Another possible factor contributing to the therapy-induced increase in mitochondrial respiration could be a shift in PBMC subpopulations toward cell types that rely more on mitochondrial oxidative phosphorylation (OXPHOS) than glycolysis for ATP production, as previously hypothesized [14]. However, this remains speculative since differential blood counts were not analyzed in the study cohort. Nonetheless, clinical data indicate a transient decrease in both leukocyte count and hemoglobin concentration, highlighting the significant impact of chemo- and radiotherapy on blood cell viability and proliferation.

We previously demonstrated that plasma levels of pro-inflammatory cytokines transiently increase in BC patients following chemo- and radiotherapy, returning to pre-treatment levels within the first year [5]. This pro-inflammatory state together with the heightened oxygen demand in PBMCs, may suggest immune cells activation after chemo- and radiotherapy treatment completion. Interestingly, previous research has shown significantly increased mitochondrial respiration in PBMCs from asthmatic patients experiencing severe exacerbation compared to healthy controls [19]. In this study, it was also speculated that this increase was linked to increased immune activity. In the present study, however, we found no significant correlations between PBMC mitochondrial respiration capacity and plasma CRP levels in the patients. Associations between PBMC respiration and other markers of inflammatory or metabolic health would be relevant to establish in future studies. Since chronic inflammation influences cancer progression, chemotherapy-induced inflammation may impact treatment outcomes and long-term risks like metabolic syndrome [20]. Notably, markers like the neutrophil-to-eosinophil ratio show prognostic value for survival in cancer patients before treatment [21]. Whether PBMC respiration holds similar potential in BC patients remains unclear.

In a study of human leukocyte cell lines, incubation with the anthracycline chemotherapy drugs doxorubicin and daunorubicin led to immediate impairment of mitochondrial respiration, while the alkylating agent cisplatin did not affect mitochondrial respiration at clinically relevant concentrations [22]. Additionally, radiotherapy has been suggested to negatively impact mitochondrial OXPHOS through damage and deletions of mitochondrial DNA (mtDNA), resulting in disturbances in the ETS [23]. Our study population only included five patients who did not receive radiotherapy and five patients who did not receive ant-estrogen therapy, limiting our ability to thoroughly investigate the impact of radiotherapy and anti-estrogen therapy on PBMC mitochondrial respiration.

Previous studies have shown that tumor-secreted factors can contribute to mitochondrial dysregulation even before initiation of treatment [24, 25]. In our study, we did not observe any differences in PBMC mitochondrial respiration between patients and controls or between patients with and without a breast tumor present at the time of pre-treatment blood sampling. Notably, we found significantly higher mitochondrial respiration after treatment in patients who received neoadjuvant chemotherapy compared to those who received adjuvant chemotherapy. This difference may partly be explained by a borderline significantly higher mitochondrial content in the neoadjuvant group post-treatment. Additionally, healthy controls had a higher mitochondrial content than BC patients before chemo- and radiotherapy, yet their PBMC respiration levels remained comparable to those of BC patients.

Chemotherapy has previously been shown to influence mitochondrial respiration in skeletal muscle from BC patients, but in contrast to our results, these studies reported either no change [11] or a decrease [8, 9] in mitochondrial respiration. PBMCs are increasingly recognized as biomarkers for bioenergetic dysfunction due to their interaction with various tissues and exposure to nutritional, metabolic, and immunological stimuli. Notably, if PBMCs reflect conditions in other tissues, they could offer a less invasive alternative to tissue biopsies. Our results suggest that mitochondrial respiration in PBMCs is not indicative of mitochondrial respiration in skeletal muscle. This aligns with the findings of a previous study, which emphasized that associations between mitochondrial respiration in long-lived and short-lived cells should be interpreted with caution [26].

The study strengths include the uniquely collected longitudinal samples of our study cohort, and the addition of healthy controls to explore the impact of BC diagnosis on PBMC mitochondrial respiration. Limitations include treatment regimen heterogeneity, insufficient statistical power to separate treatment effects, and the lack of PBMC subpopulation analysis. Additionally, the clinical significance of mitochondrial changes in PBMCs remains unclear.

In conclusion, our study demonstrates that chemo- and radiotherapy induce a transient increase in mitochondrial respiration in PBMCs from BC patients, which returns to pre-treatment levels within six months after therapy completion. Although mitochondrial content did not change significantly after treatment, it may still contribute to changes in mitochondrial respiration. Notably, the temporary increase in oxygen demand in PBMCs following chemo- and radiotherapy could reflect heightened activation of mononuclear immune cells in BC patients. An enhanced immune defense capacity, although temporary, may be beneficial for combating potential infections post-chemotherapy. On the other hand, increased immunological activity of monocytes could also lead to inflammation (e.g., macrophage infiltration and associated cytokine release) in tissues such as adipose tissue, thereby elevating the risk of inflammatory and metabolic diseases in BC survivors. The potential clinical implications of the transient increase in PBMC mitochondrial respiration warrant further investigation.

## Supplementary Information

Below is the link to the electronic supplementary material.

Supplementary file 1 (DOCX 236 KB)

## Data Availability

The data supporting the findings of this study are available from the corresponding author upon reasonable request.
