# Supplementary material for: Transient increase in mitochondrial respiration in blood cells from breast cancer patients following chemo- and radiotherapy
Source: Clin Exp Med. 2025 May 8;25(1):142. doi: 10.1007/s10238-025-01665-4 (PMC12062163; doi:10.1007/s10238-025-01665-4)
Supplement: Supplementary file 1 — (DOCX 236 KB) [file 10238_2025_1665_MOESM1_ESM.docx]

Supplementary Data for the article:

**Transient Increase in Mitochondrial Respiration in Blood Cells from Breast Cancer Patients Following Chemo- and Radiotherapy**

Marie-Louise Abrahamsen, Ida Bager Christensen, Linda Laizāne, Haboon Ismail Ahmed, Kristian Buch-Larsen, Djordje Marina, Michael Andersson, Peter Schwarz, Flemming Dela, Linn Gillberg

**Correspondence**

Linn Gillberg, [linn.gillberg@sund.ku.dk](mailto:linn.gillberg@sund.ku.dk), Department of Biomedical Sciences, University of Copenhagen, Blegdamsvej 3B, 2200 Copenhagen, Denmark

<https://orcid.org/0000-0003-0588-1663>

|  | Number of patients (percentage) |
| --- | --- |
| Malignancy grade |  |
| I | 8 (24%) |
| II | 20 (59%) |
| III | 6 (18%) |
| Surgery type |  |
| Mastectomy | 10 (29%) |
| Lumpectomy | 24 (71%) |
| Lymph node involvement |  |
| 0 | 9 (26%) |
| 1-3 | 24 (71%) |
| 4+ | 1 (3%) |
| Estrogen receptor status |  |
| Positive | 29 (85%) |
| Negative | 5 (15%) |
| HER2 status |  |
| Positive | 14 (41%) |
| Negative | 20 (59%) |
| Chemotherapy |  |
| Cyclophosphamide | 30 (88%) |
| Paclitaxel | 29 (85%) |
| Epirubicin | 26 (76%) |
| Docetaxel | 5 (15%) |
| Other | 2 (6%) |
| Radiotherapy |  |
| Yes | 29 (85%) |
| No | 5 (15%) |
| Endocrine treatment (aromatase inhibitors) |  |
| Yes | 29 (85%) |
| No | 5 (15%) |
| HER2-directed treatment (trastuzumab) |  |
| Yes | 14 (41%) |
| No | 20 (59%) |
| Anti-resorptive treatment (Zoledronic Acid or Denosumab) |  |
| Yes | 34 (100%) |
| No | 0 (0%) |

**Supplementary Table 1: Disease and treatment characteristics of the included BC patients.**

Data is presented as the number of patients (n=34 in total) and percentage of the cohort in parentheses.

*Abbreviations: ER: estrogen receptor; HER2: human epidermal growth factor receptor 2.*

|  | **Controls** | **Pre-treatment** | **Post-treatment** | **% change**  pre vs post | **6**  **months** | **% change**  post vs 6 m | **12 months** |
| --- | --- | --- | --- | --- | --- | --- | --- |
| **Intact cells** |  |  |  |  |  |  |  |
| Endogenous | 9.6 ± 2.2 | 10.2 ± 3.6 | 13.5 ± 5.2* | **+32%** | 10.5 ± 4.3¤ | **-22%** | 10.0 ± 3.2 |
| Proton leak | 3.2 ± 0.9 | 3.5 ± 2.3 | 4.1 ± 1.5 | **+17%** | 3.5 ± 2.0 | **-15%** | 3.2 ± 1.4 |
| ETS | 14.1 ± 3.5 | 14.7 ± 4.7 | 19.5 ± 8.1* | **+33%** | 15.0 ± 4.7¤ | **-23%** | 17.3 ± 6.2 |
| Non-mitochondrial | 0.8 ± 0.4 | 0.9 ± 1.3 | 0.8 ± 0.5 | **-11%** | 0.7 ± 0.6 | **-13%** | 0.7 ± 0.5 |
| **Permeabilized cells** |  |  |  |  |  |  |  |
| Endogenous | 9.6 ± 1.8 | 10.8 ± 3.5 | 14.2 ± 5.3* | **+31%** | 10.6 ± 4.1¤ | **-25%** | 9.7 ± 3.4¤ |
| LEAK_CI_ | 2.6 ± 1.0 | 2.4 ± 1.6 | 4.3 ± 3.2* | **+79%** | 2.4 ± 2.1 | **-44%** | 3.5 ± 2.6 |
| CI_p_ | 7.7 ± 2.0 | 7.0 ± 3.3 | 10.0 ± 5.8 | **+43%** | 6.9 ± 3.5 | **-31%** | 8.4 ± 3.9 |
| CI + II_p_ | 20.4 ± 4.9 | 19.8 ± 7.2 | 25.5 ± 9.8* | **+29%** | 19.6 ± 7.5¤ | **-23%** | 20.4 ± 6.5 |
| ETS | 21.6 ± 5.5 | 21.3 ± 7.0 | 27.6 ± 10.9* | **+30%** | 20.5 ± 8.0¤ | **-26%** | 23.2 ± 8.4 |
| Non-mitochondrial | 0.8 ± 0.4 | 0.9 ± 0.4 | 1.0 ± 0.7 | **+11%** | 0.8 ± 0.8 | **-20%** | 1.1 ± 1.2 |

**Supplementary Table 2: Mitochondrial O_2_ flux in** **intact and permeabilized PBMCs from BC patients and healthy controls.**

Mitochondrial O_2_ flux [pmol/(s × million PBMCs)] in PBMCs from healthy controls (n=19-20) and BC patients pre (n=32), post (n=30), 6 months (n=28), and 12 months (n=28-30) after chemo- and radiotherapy. Data is presented as mean ± standard deviation. Endogenous: Endogenous routine respiration (no substrates, inhibitors, or permeabilization reagent added). Proton leak: Oxygen is consumed due to a proton leak over the inner mitochondrial membrane. ETS: Maximal capacity of the electron transfer system. Non-mitochondrial: non-mitochondrial respiration. LEAK_CI_: Leak respiration with the presence of complex I-linked substrates. CI_P_: Complex I-linked respiration. CI+CII_P_: Complex I + II-linked respiration. The percentage change was calculated between visits: pre-treatment vs. post-treatment and post-treatment vs. six months. Unpaired t-tests were done between patients pre-treatment and controls. Data from the four different patient visits was analyzed using mixed effects analysis with the Geisser-Greenhouse correction for variance in combination with Tukey’s multiple comparisons test. *: significantly different from pre-treatment (p-value<0.05), ¤: significantly different from post-treatment (p-value<0.05).

*Abbreviations: BC: breast cancer, ETS: electron transfer system, PBMCs: peripheral blood mononuclear cells.*

|  | **CRP (mg/L)** |
| --- | --- |
| **Intact cells** |  |
| Endogenous | r=-0.07, p=0.74 |
| Proton leak | r=-0.06, p=0.77 |
| ETS | r=0.01, p=0.95 |
| Non-mitochondrial | r=-0.10, p=0.95 |
| **Permeabilized cells** |  |
| Endogenous | r=-0.08, p=0.70 |
| LEAK_CI_ | r=-0.23, p=0.23 |
| CI_p_ | r=-0.06, p=0.75 |
| CI + II_p_ | r=0.04, p=0.86 |
| ETS | r=-0.07, p=0.73 |
| Non-mitochondrial | r=0.60, p=0.42 |

**Supplementary Table 3: Correlations between mitochondrial respiration in PBMCs and plasma CRP levels in BC patients pre-treatment and healthy controls.**

Results are presented as Spearman correlation coefficients (r) and p-values (p). Endogenous: Endogenous routine respiration (no substrates, inhibitors, or permeabilization reagent added). Proton leak: Oxygen is consumed due to a proton leak over the inner mitochondrial membrane. ETS: Maximal capacity of the electron transfer system. Non-mitochondrial: non-mitochondrial respiration. LEAK_CI_: Leak respiration with the presence of complex I-linked substrates. CI_P_: Complex I-linked respiration. CI+CII_P_: Complex I + II-linked respiration. CRP values have previously been published (Lindholm et al. 2024) and were available for a subgroup of controls (n=7) and BC patients pre-treatment (n=24). *Abbreviations: BC: breast cancer, CRP, C-reactive protein, ETS: electron transfer system, PBMCs: peripheral blood mononuclear cells.*

|  | **Mitochondrial content**  (mtDNA/ncDNA) |
| --- | --- |
| **Intact cells** |  |
| Endogenous | r=0.16, p=0.25 |
| Proton leak | r=0.29, **p=0.04** |
| ETS | r=-0.01, p=0.94 |
| Non-mitochondrial | r=0.37, **p=0.03** |
| **Permeabilized cells** |  |
| Endogenous | r=0.25, p=0.09 |
| LEAK_CI_ | r=0.26, p=0.06 |
| CI_p_ | r=0.29, **p=0.04** |
| CI + II_p_ | r=-0.07, p=0.62 |
| ETS | r=-0.10, p=0.50 |
| Non-mitochondrial | r=0.35, **p=0.045** |

**Supplementary Table 4: Correlations between mitochondrial respiration and content in PBMCs from BC patients pre-treatment and healthy controls.**

Results are presented as Spearman correlation coefficients (r) and p-values (p) for the correlation. Endogenous: Endogenous routine respiration (no substrates, inhibitors, or permeabilization reagent added). Proton leak: Oxygen is consumed due to a proton leak over the inner mitochondrial membrane. ETS: Maximal capacity of the electron transfer system. Non-mitochondrial: non-mitochondrial respiration. LEAK_CI_: Leak respiration with the presence of complex I-linked substrates. CI_P_: Complex I-linked respiration. CI+CII_P_: Complex I + II-linked respiration. *Abbreviations: BC: breast cancer, ETS: electron transfer system, PBMCs: peripheral blood mononuclear cells.*

**Supplementary Figure 1: Overview of samples and data in mitochondrial respiration analysis.**

In the current study, 34 BC patients were included and followed at four different visits: pre-treatment, post-treatment, six and twelve months after treatment. In addition to the BC patients 20 healthy controls were included for one visit. However, all data was not available for all participants at all visits. In some cases, the participants missed their visit and in other cases, the data was excluded due to errors or technical issues. In the respiration protocol for permeabilized cells (left), there were pre: n=32, post: n=30, 6 months: n=28, 12 months: n=30, controls: n=19. In the respiration protocol for intact cells (right), there were pre: n=32, post: n=30, 6 months: n=28, 12 months: n=28, controls: n=20.

**
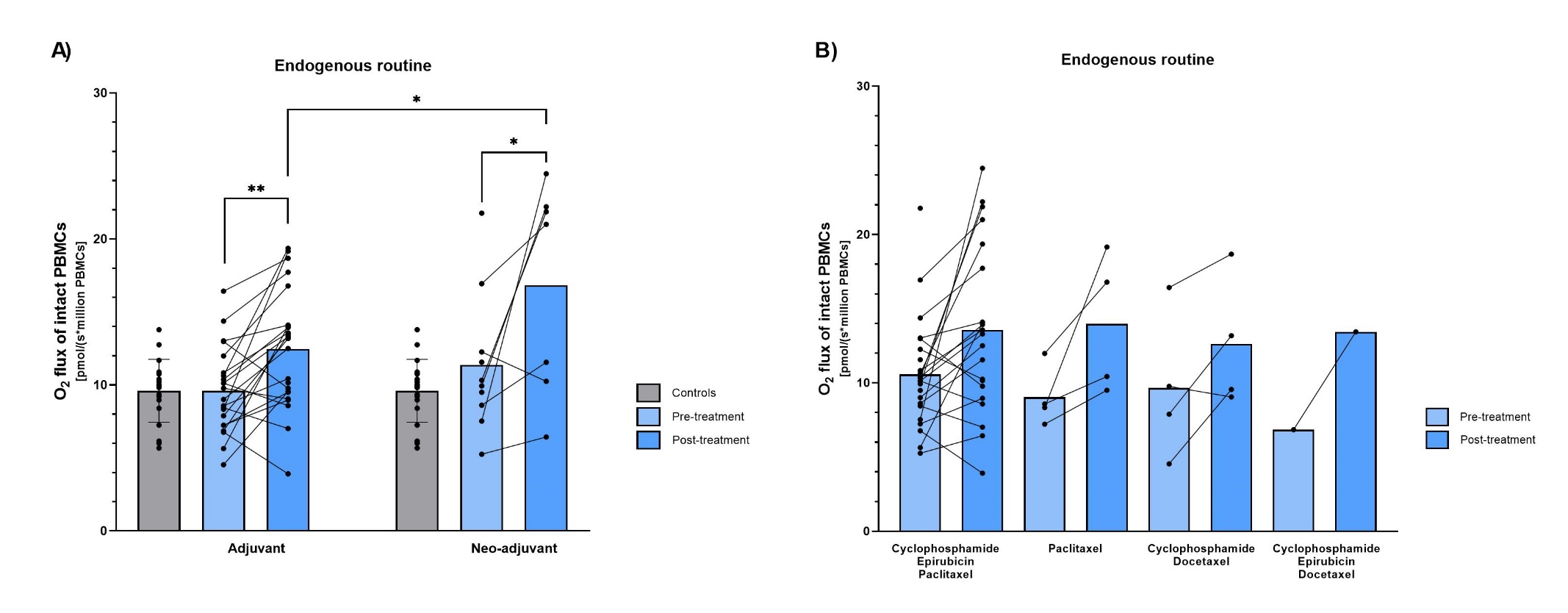
**

**Supplementary Figure 2: Comparisons of endogenous routine respiration in intact PBMCs between treatment groups.**

**A)** Mitochondrial O_2_ flux [pmol/(s × million PBMCs)] of intact PBMCs from controls (n=20) and BC patients receiving adjuvant chemotherapy (after surgery, n=24) vs neo-adjuvant chemotherapy (before surgery, n=10) pre- and post-treatment. The top of the bar represents the mean, whereas the error bar shows the standard deviation. Endogenous: Endogenous routine respiration (no substrates or inhibitors added). Unpaired t-tests were done between the two treatment groups, whereas paired t-test was done within each group between pre-and post-treatment visits. **: p-value < 0.005, *: p-value < 0.05. **B)** Mitochondrial O_2_ flux [pmol/(s × million PBMCs)] of intact PBMCs before and after treatment from BC patients receiving Cyclophosphamide, Epirubicin, and Paclitaxel (n=23), Paclitaxel (n=4), Cyclophosphamide and Docetaxel (n=4), and Cyclophosphamide, Epirubicin, and Docetaxel (n=1). Statistical analyses were not performed due to low statistical power. *Abbreviations: BC: breast cancer, PBMCs: peripheral blood mononuclear cells.*
